# RUNX3/H3K27me3 Co-Expression Defines a Better Prognosis in Surgically Resected Stage I and Postoperative Chemotherapy-Naive Non-Small-Cell Lung Cancer

**DOI:** 10.1155/2022/5752263

**Published:** 2022-03-24

**Authors:** Xiaohui Chen, Yujie Deng, Chuanzhong Huang, Yi Shi, Jianping Lu, Guibin Weng, Weifeng Zhu, Kunshou Zhu, Junqiang Chen, Jiancheng Li

**Affiliations:** ^1^Department of Thoracic Surgery, Fujian Medical University Cancer Hospital, Fujian Cancer Hospital, Fuzhou, Fujian 350014, China; ^2^College of Clinical Medicine for Oncology, Fujian Medical University, Fuzhou, China; ^3^Graduate School of Fujian Medical University, Fuzhou, China; ^4^Department of Medical Oncology, The First Affiliated Hospital of Fujian Medical University, Fuzhou, Fujian 350005, China; ^5^Laboratory of Immuno-Oncology, Fujian Medical University Cancer Hospital, Fujian Cancer Hospital, Fuzhou, Fujian 350014, China; ^6^Department of Pathology, Fujian Medical University Cancer Hospital, Fujian Cancer Hospital, Fuzhou, Fujian 350014, China; ^7^Department of Radiation Oncology, Fujian Medical University Cancer Hospital, Fujian Cancer Hospital, Fuzhou, Fujian 350014, China

## Abstract

The purpose of this study is to investigate the significance of RUNX3/H3K27me3 co-expression in surgically resected non-small-cell lung cancer (NSCLC) patients. Using tissue microarray (TMA), immunohistochemistry, fluorescent double immunostaining, and western blotting, 208 NSCLC and 5 benign pulmonary patients were studied of their expression of runt-related transcription factor 3 (RUNX3), trimethylated histone H3 at lysine 27 (H3K27me3), enhancer of zeste homolog 2 (EZH2), and Ki-67. Apoptotic index in cancerous tissue was evaluated via TdT-mediated dUTP-biotin nick end labeling (TUNEL). The correlation between clinicopathologic parameters and overall survival was determined by Cox regression and Kaplan–Meier survival estimates and log-rank test. GEPIA and KM plotter were used for validation of some survival analyses. As a result, together with other regular prognostic factors, RUNX3/H3K27me3 co-expression was found to be closely correlated with better prognosis in either pTNM-I or POCT-naive NSCLC patients, which might partially result from a higher cancerous apoptotic index. In conclusion, RUNX3/H3K27me3 co-expression defined some specific NSCLC population with better prognosis and longer OS and could probably be used as a biomarker in the prediction of better postoperative outcomes.

## 1. Introduction

Lung cancer is still the leading cause of cancer-related deaths in both sexes in China and worldwide [1, 2], and non-small-cell lung cancer (NSCLC) accounts for nearly 85% of all cases. For decades, diagnosis of lung cancer occurred so late that nearly two-thirds of patients had lost the opportunity for radical resection. Recently, with the prevalence of latest diagnostic techniques like low-dose computed tomography (LDCT) or artificial intelligence (AI) and use of liquid biopsy in lung cancer screening, more and more early-stage cases had been diagnosed and received timely surgical resection [3–6]. Although many surgically resected patients shared identical histology and pathologic stage, their prognosis varied a lot, with nearly one-third of pTNM-stage I patients suffering from early postoperative relapse or distant metastasis [7, 8]. Based on this context, molecular staging with sensitive biomarkers on the purpose of accurate prediction of different likelihood of early relapse or metastasis is necessary. One major discrepancy underlying the different prognoses for patients with the same pathologic stage is their different epigenetic status, mainly recognized as histone modifications or DNA methylation involved in the regulation of various oncogenes or tumor suppressor genes (TSGs), facilitating tumorigenesis and/or progression of different types of human malignancies. One such histone modification, trimethylated histone H3 at lysine 27 (H3K27me3), catalyzed by enhancer of zeste homolog 2 (EZH2) and functioning as a mark for de novo DNA methylation in cancer cells by recruitment of DNA methyltransferases (DNMTs), is usually recognized as a transcription-suppressive histone modification and proven to be involved in cell cycle progression and proliferation regulation, as well as hypermethylation of tumor suppressor molecules like mediating epigenetic silencing of a well-known TSG—human runt-related transcription factor 3 (RUNX3) in a great subset of cancers [9–14].

RUNX3, a remarkable biomarker demonstrated in many solid malignancies, plays a main role of tumor suppression and interacts with other signaling molecules in the context of carcinogenesis in various cancer types including NSCLC [15–23, 15]. Loss of expression and cytoplasmic mislocalization had been indicated partly as underlying causes of RUNX3 dysfunction in many cancer types.

Although loss of RUNX3 expression is prevailing in human solid malignancies, the role of its association in pathogenesis of respiratory malignancies still requires further elucidation. Our previous studies firstly found that NSCLC patients with higher trimethylated histone H3 at lysine 27 (H3K27me3) level demonstrated a lower probability of postoperative local relapse and determined loss of H3K27me3 expression as an independent risk factor [24, 25], and secondly found that NSCLC patients with higher RUNX3 level demonstrated a lower likelihood of postoperative distant metastasis, and the loss of RUNX3 expression predicted worst outcome and shorter overall survival (OS) [26]. In light of both local relapse and distant metastasis contributing to worse outcome in the postoperative setting and the epigenetic regulation that histone modification might have on oncogenes and TSGs, we thus hypothesized that assessment of H3K27me3/RUNX3 co-expression might probably play some role in predicting the surgical outcome of NSCLC patients after radical resection.

In the present study, we sought to investigate the potential interrelationship of expression of RUNX3, H3K27me3 and its methyltransferase EZH2 in NSCLC patients, their correlation with clinicopathologic parameters, and the prognostic significance as well, finding that expression of RUNX3 was closely correlated with H3K27me3 and their co-expression was significantly associated with better prognosis especially in either stage I or postoperative chemotherapy-naive (POCT-naive) NSCLC patients.

## 2. Materials and Methods

### 2.1. Patients and Tissue Samples

Archival formalin-fixed, paraffin-embedded tissue sections from 5 normal lung tissues (with a diagnosis of pulmonary bulla) and 208 patients who underwent surgery for NSCLC at the Department of Thoracic Surgery of Fujian Medical University Cancer Hospital during 2010-2011 were selected. None had received chemo or radiotherapy prior to tissue collection. The histopathologic features of cancerous specimens and TNM staging were determined according to the 8^th^ version of AJCC guidelines for NSCLC. The final follow-up date was February 5, 2020, and all patients were available of their survival data. Patients' survival data were censored if they were still alive or dead of disease other than lung cancer at the date of surveillance. The study protocol was approved by the Human Ethics Review Committee of Fujian Medical University Cancer Hospital, and signed informed consent was obtained from each patient.

### 2.2. Tissue Microarray Building

A fresh section was cut from each donor block, stained with hematoxylin and eosin (HE), and used as a guide to select the morphologically most-representative regions of the tumor to sample the individual core needle biopsies. A duplicate of 0.6 mm diameter cores were then punched from tumoral areas of each donor tissue block and introduced into previously prepared recipient paraffin blocks [27], after having made hosting holes in the blocks with a tissue microarrayer (Beecher Instruments, Silver Spring, MD). We constructed 4 recipient blocks with a maximum of 10 × 6 dots. With a microtome, 4 *μ*m sections were cut from the TMA blocks and placed onto 3-aminopropyltriethoxysilane-coated glass slides to generate TMA slides for further analyses. Some sections were stained with HE in a routine manner for histological examination.

### 2.3. Immunohistochemical Detection of RUNX3, H3K27me3, EZH2, and Ki-67

Immunohistochemistry was performed with the indirect enzyme-labeled antibody method, as described previously [24–26]. Information of antibodies used is shown in Table 1. For detection of RUNX3, H3K27me3, and EZH2, TMA sections were deparaffinized with toluene and rehydrated in graded alcohols. After autoclaved for 15 min at 120°C in 10 mM citrate buffer (pH 6.0) for antigen retrieval, endogenous peroxidase was inactivated with 0.3% hydrogen peroxide in methanol for 15 min. The sections were then preincubated with 500 *μ*g/ml normal goat IgG dissolved in 1% BSA in PBS (pH 7.4) for 1 h, reacted with primary antibodies for 16 h, washed with 0.075% Brij 35 in PBS, and then incubated with HRP-conjugated goat anti-mouse (RUNX3)/rabbit IgG (H3K27me3/EZH2/Ki-67) in 1% BSA in PBS for 1 h. After washing with 0.075% Brij 35 in PBS, the sites of HRP were visualized with DAB and H_2_O_2_. As a negative control, some sections were reacted with normal mouse/rabbit IgG instead of the specific antibodies. For simultaneous fluorescent double immunostaining of H3K27me3 and RUNX3, the sections were incubated with Alexa 546 anti-rabbit IgG and Alexa 488 anti-mouse IgG (both 1 : 500) in darkness for 1 h, then washed with 0.075% Brij 35 in PBS in darkness, and finally observed with 0.5 *μ*g/ml DAPI for 1 min. The stained slides were analyzed under a laser scanning microscope (LSM 5 PASCAL; Carl Zeiss Inc., Germany).

### 2.4. Validation via RNA Sequencing Expression and Survival Analyses of Core Genes

The gene expression profiling interactive analysis (GEPIA) (http://cancer-pku.cn) was applied to analyze the data of RNA sequencing expression on the basis of samples from the GTEx projects and TCGA. Kaplan-Meier plotter (http://kmplot.com/analysis/index.php?p=service&cancer=lung) was used to determine the effect of genes (RUNX3 and EZH2) on survival based on EGA, TCGA database, and GEO (Affymetrix microarrays only). Survival within groups was compared by log-rank estimates.

### 2.5. Western Blotting Analyses of RUNX3, H3K27me3, EZH2, and *β*-Actin

Western blotting was carried out as detailed previously [24]. In brief, 7 pairs of human NSCLC specimens together with their related normal lung tissue were used in this session, and each procedure was repeated 3 times. The specimens were homogenized, and the lysates were centrifuged. Soluble proteins were separated on 10% SDS-PAGE gel (Daiichi Pure Chemical, Tokyo, Japan) with equal amounts (10 *μ*g) of protein per lane. Separated proteins were electrophoretically transferred onto polyvinylidene difluoride (PVDF) membranes (Millipore Corporation, MA, USA), blocked with 10% nonfat milk in TBS (20 mM Tris buffer, pH 7.6, and 150 mM NaCl) for 1 h and then incubated overnight at 4°C with EZH2, H3K27me3, RUNX3, and *β*-actin antibodies. As a secondary antibody, HRP-goat anti-rabbit IgG was reacted for 1 h and then the bands were visualized with DAB, Ni, Co, and H_2_O_2_. The grey ratio value of EZH2/H3K27me3/RUNX3 to *β*-actin for each specimen was calculated. Individual value of EZH2/H3K27me3/RUNX3 to *β*-actin for each patient was expressed as mean ± SEM, and then final value in normal and cancerous lung tissue was compared.

### 2.6. TUNEL Staining for Apoptotic Cells in NSCLCs

To identify nuclei with DNA strand breaks at a cellular level, TUNEL was performed according to the method of Gavrieli et al. [28], with a slight modification. Detailed procedure was described previously [26]. For statistical analysis, more than 10,000 cancer cells/patient were counted, and the number of TUNEL-positive cells was expressed per 1000 of the total cells (mean ± SEM). Data for different groups were compared for statistical difference using Student's *t*-test. A *P* value of <0.05 denoted the presence of a significant difference.

### 2.7. Statistical Analysis

The best cutoffs of RUNX3 and H3K27me3 were determined via X-tile software program by dichotomizing them into high and low expression subgroups, as described in our previous studies [24–26]. The SPSS 24.0 statistical software package (SPSS Inc, Chicago, IL, USA) and GraphPad Prism (Version 8.3.0) were employed for all analyses. The association between tested markers and different clinicopathologic parameters of the patients were evaluated by Pearson's *χ*^2^ or Fisher's exact test as appropriate. Survival functions were estimated using the Kaplan–Meier method and compared using log-rank test. The Cox proportional hazard model was used to evaluate the association between various markers and patient's survival. Univariate and multivariate analyses were determined by Cox regression. A 2-sided *P* value less than 0.05 was considered statistically significant.

## 3. Results

### 3.1. Clinicopathologic Data of Patients

Among the 208 TMA cancerous tissue dots, 20 dropped off and 188 were left over (188/208, 90.4%). As shown in Table 2, the leftover NSCLC included 128 males and 60 females and had a mean age of 58 years old. By histological classification, 75 cases were LUSC and 113 were LUAD. In the LUSC group, the well, moderately, and poorly differentiated numbers were 4, 57, and 14, respectively. In the LUAD group, the predominant growth pattern numbers for lepidic, acinar, papillary, micropapillary, and solid were 10, 75, 10, 1, and 17, respectively. According to Xie et al.'s report [29], we divided the BMI into 2 categories, i.e., ① ≤20.3 kg/m^2^ and ② >20.3 kg/m^2^. The number for these 2 categories of BMI in LUAD was 17 and 96, and for LUSC, the number was 13 and 62, respectively. As for ECOG score, the number of “≤1” and “>1” in LUAD and LUSC was 86 and 27, as well as 59 and 16, respectively. In the LUAD group, 56 were smokers and 57 were nonsmokers, while in LUSC group, the number was 60 and 15. Serum CEA level was found abnormal in 55 LUAD and 19 LUSC patients. Pleural involvement (PI) was positive in 92 LUAD and 44 LUSC patients. Thirteen LUAD and 4 LUSC patients were positive of vascular invasion (VI). Fifty-four LUAD and 31 LUSC patients were positive of lymphatic vessel involvement (LVI), while others were all negative. Nerve invasion (NI) was found in 69 patients, 45 in LUAD and 24 in LUSC. For the RUNX3 localization, the number of negative, nuclear, cytoplasmic, and whole-cell patterns in LUAD and LUSC is 35, 3, 38, 37 and 20, 7, 14, 34. As for TNM staging, the number of stage I through IV was 43, 21, 43, and 6 in LUAD and 25, 20, 30, and 0 in LUSC. For T-staging, the number of stage 1a, 1b, 1c, 2a, 2b, 3, and 4 was 1, 7, 13, 58, 15, 12, and 7 in LUAD and 1, 6, 5, 24, 8, 14, and 17 in LUSC. For N-staging, the number of stage from 0 through 3 was 54, 17, 31, and 11 in LUAD and 43, 15, 15, and 2 in LUSC. For M-staging, the number of stage 0, 1a, 1b, and 1c was 107, 3, 2, and 1 in LUAD and 75, 0, 0, and 0 in LUSC. The number of different surgical procedures, i.e., sublobar resection, lobectomy, combined lobectomies, and pneumonectomy, in LUAD and LUSC subgroups is 5, 92, 12, 4 and 0, 52, 12, 11, respectively. VATS was used in 52 patients in LUAD and 13 in LUSC group. Twenty-three LUAD and 14 LUSC patients received postoperative radiotherapy (PORT). Fifty-seven LUAD and 41 LUSC patients received postoperative chemotherapy (POCT). Nineteen LUAD and 13 LUSC patients received postoperative chemoradiotherapy (POCRT). Postoperative regional relapse was present in 27 LUAD and 20 LUSC patients, while postoperative distant metastasis was found in 43 LUAD and 24 LUSC patients. Postoperative follow-up data were available for all patients, and the average follow-up time in LUAD and LUSC groups was 79.5 and 81.0 months, respectively.

### 3.2. Expression of RUNX3, H3K27me3, and EZH2 in NSCLC Tissues and Their Correlation with Clinicopathologic Variables and Survival

#### 3.2.1. RUNX3

**Figure 1:**
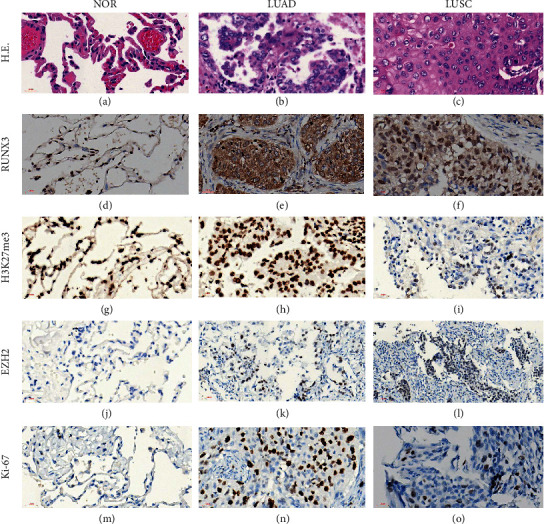
Immunostaining for RUNX3, H3K27me3, EZH2, and Ki-67 in normal lung tissue, LUAD, and LUSC. (a–c) HE staining. (d–f) RUNX3 staining. (g–i) H3K27me3 staining. (j–l) EZH2 staining. (m–o) Ki-67 staining (magnification, ×400; scale bar = 20 *μ*m).

**Figure 2:**
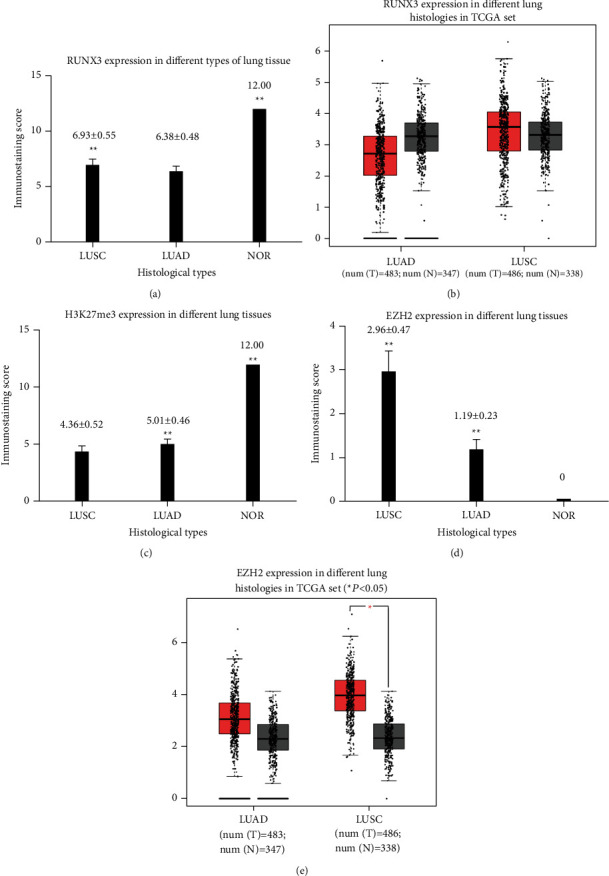
Immunostaining score in normal lung tissue, LUAD, and LUSC (data expressed in mean ± SEM). (a) RUNX3 expression in different lung tissue, ^*∗∗*^*P* < 0.01. (b) GEPIA analysis on different expression of RUNX3 in normal lung, LUSC, and LUAD, demonstrating no statistical difference among them. (c) H3K27me3 expression in different lung tissue, ^*∗∗*^*P* < 0.01. (d) EZH2 expression in different lung tissue, ^*∗∗*^*P* < 0.01. (e) GEPIA analysis on different expression of EZH2 in normal lung, LUSC, and LUAD, “^*∗*^” means *P* < 0.05.

Typical normal lung tissue, LUAD, and LUSC were stained in HE to demonstrate the normalcy of used tissue (Figures 1(a)–1(c)). Cancerous samples were categorized into low (IHC score ≤3) and high (IHC score >3) expression subgroups for RUNX3 expression based on a cutoff point determined in our previous studies [26]. As shown in Figures 1(d)–1(f) and 2(a), the immunostaining score of RUNX3 was significantly higher in normal lung compared to LUSC or LUAD (12.00 vs. 6.93 ± 0.55 vs. 6.38 ± 0.48, ^*∗∗*^*P* < 0.01), while GEPIA analyses demonstrated no discrepancy in RUNX3 expression in either LUSC or LUAD when compared to their paired normal lung tissue (Figure 2(b)).

As indicated in Table 3, higher RUNX3 expression was significantly associated with aged patients (*P*=0.025), lower ECOG PS (*P*=0.019), absence of postoperative distant metastasis (*P*=0.002), higher H3K27me3 expression (*P*=0.001), and higher EZH2 expression (*P*=0.018). No association had been discovered between expression of RUNX3 and gender, histology, smoking status, BMI, LN involvement, lymphatic vessels invasion, nerve invasion, pleural invasion, vascular invasion, T-staging, mediastinal LN involvement, TNM staging, degree of resectibility, depth of invasion, serum CEA level, or postoperative regional relapse (all *P* > 0.05).

#### 3.2.2. H3K27me3

Calculated staining score of immunopositive cells and cutoff value determination were mentioned in our previous studies [24, 25]. Expression of H3K27me3 in cancerous samples was categorized into low (IHC score ≤4) and high (IHC score >4) subgroups. Of 188 cases, 116 (62%) were of high H3K27me3 expression while 72 (38%) were of low expression. As shown in Figure 2(c), the staining score of H3K27me3 was significantly higher in normal lung tissue compared to LUSC or LUAD (12.00 vs. 4.36 ± 0.52 vs. 5.01 ± 0.46, ^*∗∗*^*P* < 0.01).

Correlation of H3K27me3 expression and clinicopathologic parameters was determined by *χ*^2^ analysis (Table 3), indicating that lower H3K27me3 expression was significantly associated with male gender (*P*=0.010), higher ECOG PS (*P* < 0.001), smokers (*P* < 0.001), non-stage-I disease (*P*=0.030), postoperative regional relapse (*P*=0.006), lower RUNX3 expression (*P*=0.001), non-nuclear RUNX3 localization (*P*=0.003), and lower EZH2 expression (*P*=0.002), while no association had been observed between H3K27me3 expression and age, histological types, BMI, lymphatic vessels invasion, nerve invasion, vascular invasion, pleural invasion, T-staging, LN involvement, mediastinal LN involvement, M-staging, degree of resectibility, depth of invasion, serum CEA level, postoperative metastasis, or Ki-67 expression level (all *P* > 0.05).

#### 3.2.3. EZH2

EZH2 was localized in nuclei of NSCLCs while it was negative in normal lung tissue (Figures 1(j)–1(l)). Staining score of EZH2 also ranged from 0 to 12. Using an average value of 1.9, we determined the samples into low (IHC score ≤1.9) and high (IHC score >1.9) expression subgroups for EZH2. All 5 normal lung tissues were negative of EZH2 expression. Of 188 NSCLC cases, 63 (34%) were of high while 125 (66%) were of low expression. As shown in Figure 2(d), the staining score of EZH2 was significantly lower in normal lung tissue compared to LUSC or LUAD (0 vs. 2.96 ± 0.47 vs. 1.19 ± 0.23, ^*∗∗*^*P* < 0.01). GEPIA analysis demonstrated that no expression discrepancy in EZH2 existed in LUAD when compared to paired normal lung tissue, while EZH2 expression in LUSC was statistically higher than its paired normal lung tissue (Figure 2(e)).

Correlation of EZH2 expression and clinicopathologic parameters was determined via *χ*^2^ analysis (Table 3), showing that lower EZH2 expression was significantly associated with LUAD histology (*P*=0.005), lower RUNX3 expression (*P*=0.018), non-nuclear RUNX3 expression (*P*=0.001), lower H3K27me3 expression (*P*=0.002), and low Ki-67 level (*P* < 0.001). No association had been observed between EZH2 expression and age, gender, ECOG score, BMI, smoking status, lymphatic vessels invasion, nerve invasion, pleural invasion, vascular invasion, T-staging, LN involvement, mediastinal LN involvement (N2 disease), M-staging, TNM staging, degree of resectibility, depth of invasion, serum CEA level, postoperative regional relapse, or postoperative metastasis (all *P* > 0.05).

#### 3.2.4. RUNX3/H2K27me3 Co-Expression

**Figure 3:**
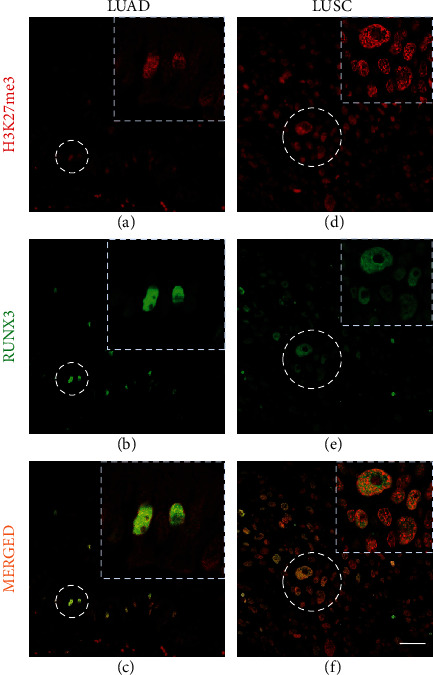
Immunofluorescent double staining for H3K27me3 (rhodamine) and RUNX3 (FITC) in LUAD (a–c) and LUSC (d–f). Highlighted dotted area indicated the presence of simultaneous high expression of both RUNX3 and H3K27me3 (scale bar = 20 *μ*m).

Co-expression of RUNX3/H2K27me3 was observed in 35.4% (40/113) LUAD and in 25.3% (19/75) LUSC patients, as was also confirmed in immunofluorescent double staining for RUNX3 and H2K27me3 (Figure 3). As shown in Table 4, absence of RUNX3/H2K27me3 co-expression was associated with male gender (*P*=0.037), higher ECOG PS (*P* < 0.001), smoker (*P*=0.001), higher probability of postoperative relapse (*P* < 0.001), higher probability of postoperative distant metastasis (*P*=0.048), non-nuclear RUNX3 localization (*P* < 0.001), and lower EZH2 expression (*P*=0.016). No association had been observed between RUNX3/H2K27me3 co-expression and age, histology, BMI, lymphatic vessels invasion, nerve invasion, pleural invasion, vascular invasion, T-staging, LN involvement, mediastinal LN involvement (N2 disease), M-staging, TNM staging, degree of resectibility, depth of invasion, serum CEA level, or Ki-67 expression (all *P* > 0.05).

### 3.3. Western Blotting of EZH2, H3K27me3, and RUNX3 in NSCLC Tissues

**Figure 4:**
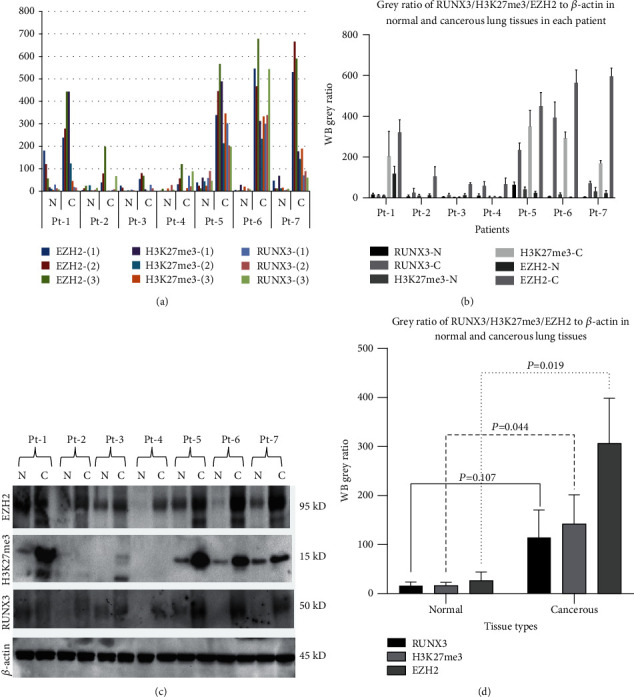
Western blotting result of 7 pairs of normal (N) and cancerous (C) lung tissue. (a) Individual grey ratio of EZH2/H3K27me3/RUNX3 to *β*-actin in 3 repeated experiments. (b) Grey ratio of EZH2/H3K27me3/RUNX3 to *β*-actin for each patient expressed in mean ± SEM. (c) Typical photograph of western blotting result for EZH2, H3K27me3, RUNX3, and *β*-actin. (d) Comparison of grey ratio of EZH2/H3K27me3/RUNX3 to *β*-actin in normal and cancerous lung tissue (difference existed in EZH2 (*P*=0.019) and H3K27me3 (*P*=0.044) but not in RUNX3 (*P*=0.107)).

Individual grey ratio for EZH2 (95 kD), H3K27me3 (15 kD), and RUNX3 (50 kD) to *β*-actin (45 kD) in 7 pairs of NSCLC and related normal lung tissues is shown in Figure 4(a). Each procedure was repeated for 3 times and the grey ratio value was recorded. Individual value for RUNX3, H3K27me3, and EZH2 was calculated and expressed as “mean ± SEM” (Figure 4(b)). Typical photograph of western blot is shown in Figure 4(c), and final comparison of RUNX3/H3K27me3/EZH2 between normal and cancerous lung tissue is indicated in Figure 4(d), demonstrating obvious statistical discrepancy on H3K27me3/EZH2 while not on RUNX3.

### 3.4. Survival Analyses

As indicated in Tables 5 and 6, univariate analysis indicated that factors favorable for longer overall survival (OS) in NSCLC patients were as follows: ECOG PS ≤1 (*P* < 0.001), no LN involvement (*P* < 0.001), no mediastinal LN involvement or absence of N2 disease (*P* < 0.001), no distant metastasis at time of diagnosis (*P* < 0.001), early TNM staging, i.e., stage I-II (*P* < 0.001) or stage I (*P* < 0.001), no pleural involvement (*P*=0.012), no lymphatic vessel involvement (*P*=0.003), no nerve involvement (*P* < 0.001), R0 resection (*P* < 0.001), employment of PORT (*P*=0.003), employment of postoperative chemoradiotherapy (*P*=0.006), normal serum CEA level at diagnosis (*P*=0.001), no postoperative regional relapse (*P* < 0.001), no postoperative distant metastasis (*P* < 0.001), higher H3K27me3 expression (*P*=0.009), and presence of RUNX3/H3K27me3 co-expression (*P*=0.003). No correlation had been observed between better survival and age, gender, smoking status, BMI, histology, T-staging, vascular invasion, postoperative chemotherapy, RUNX3 expression level, nuclear RUNX3, or EZH2 expression (all *P* > 0.05). Multivariate analyses showed that no distant metastasis at the time of diagnosis (*P*=0.027), TNM-I staging (*P*=0.036), no postoperative regional relapse (*P* < 0.001), ECOG PS ≤1 (*P* < 0.001), and no postoperative distant metastasis (*P*=0.044) were the independent prognostic factors for better OS.

**Figure 5:**
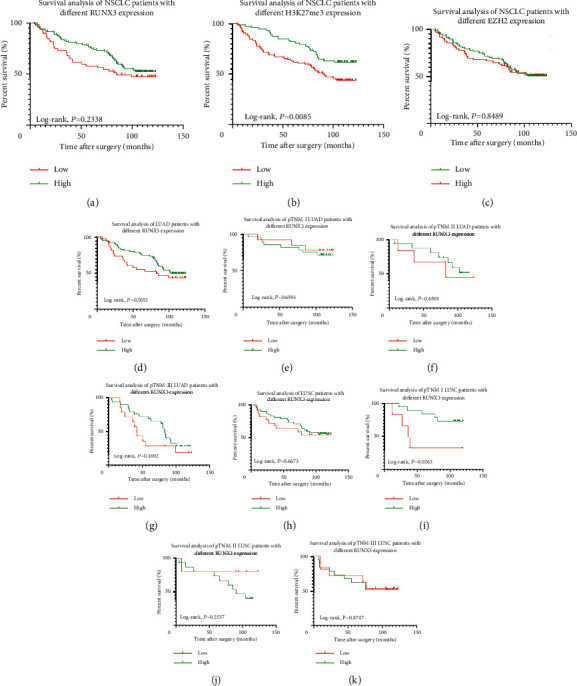
Survival analysis of the tested population. (a) Different OS in NSCLC with different RUNX3 levels (*P*=0.2338). (b) Different OS in NSCLC with different H3K27me3 levels (*P*=0.0085). (c) Different OS in NSCLC with different EZH2 levels (*P*=0.8489). (d–g) Different OS in LUAD and pTNM-stage I through III LUAD with different RUNX3 levels (all *P* > 0.05). (h) Different OS in LUSC with different RUNX3 levels (*P*=0.6673). (i) Different OS in pTNM-stage I LUSC with different RUNX3 levels (*P* = 0.0263). (j, k) Different OS in pTNM-stage II through III LUSC with different RUNX3 levels (both *P* > 0.05).

**Figure 6:**
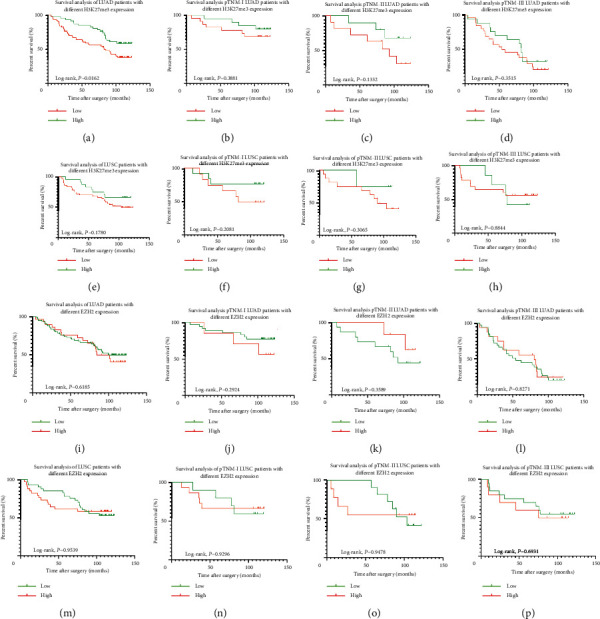
Survival analysis of the tested population. (a) Different OS in LUAD with different H3K27me3 levels (*P*=0.0162). (b–d) Different OS in pTNM-stage I through III LUSC with different H3K27me3 levels (all *P* > 0.05). (e–h) Different OS in LUSC and pTNM-stage I through III LUSC with different H3K27me3 levels (all *P* > 0.05). (i–l) Different OS in LUAD and pTNM-stage I through III LUAD with different EZH2 levels (all *P* > 0.05). (m–p) Different OS in LUSC and pTNM-stage I through III LUSC with different EZH2 levels (all *P* > 0.05).

Kaplan–Meier survival analyses with log-rank tests indicated that no difference had been demonstrated in NSCLC patients with different expression level of RUNX3 (*P*=0.2338, Figure 5(a)) or EZH2 (*P*=0.8489, Figure 5(c)), while NSCLC patients with higher expression of H3K27me3 demonstrated better prognosis in survival (*P*=0.0085, Figure 5(b)). Further stratification analyses (Figures 5(d)–5(k)) based on various RUNX3 expression levels showed that, except for a significant difference in pTNM stage-I LUSC patients (*P*=0.0263), survival benefit had been demonstrated neither in patients with different histology nor in patients with different TNM staging (all *P* > 0.05). In the case of H3K27me3 (Figures 6(a)–6(h)), however, statistical significance was found in LUAD patients with different expression levels, with better outcome in higher level patients, while no survival benefit had been found in LUSC patients with different levels of H3K27me3 expression (all *P* > 0.05). Also, the situation is similar in EZH2 (Figures 6(i)–6(p)), and no survival discrepancy had ever been found in the patient subgroups with different histology or TNM staging related to different EZH2 level (all *P* > 0.05).

**Figure 7:**
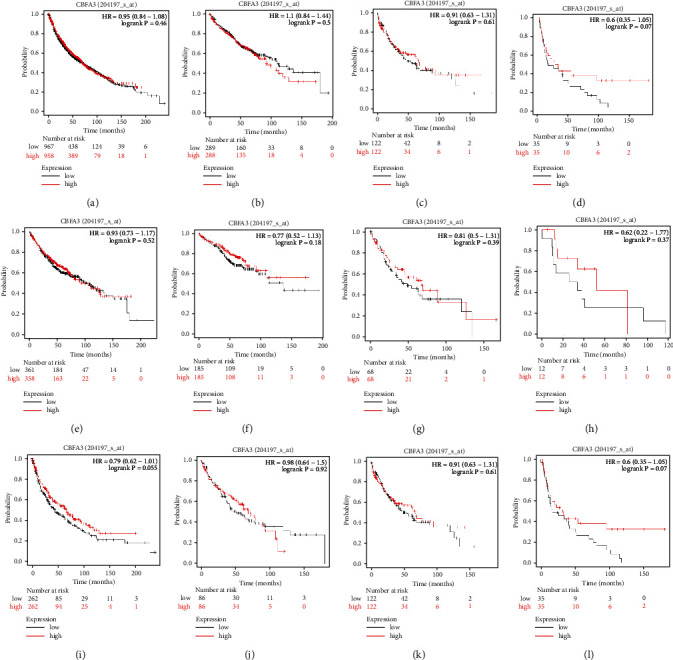
KM plotter validation of RUNX3 expression in NSCLC patients. (a–d) Different OS in NSCLC and pTNM-stage I through III NSCLC with different RUNX3 levels (all *P* > 0.05). (e–h) Different OS in LUAD and pTNM-stage I through III LUAD with different RUNX3 levels (all *P* > 0.05). (i–l) Different OS in LUSC and pTNM-stage I through III LUSC with different RUNX3 levels (all *P* > 0.05).

**Figure 8:**
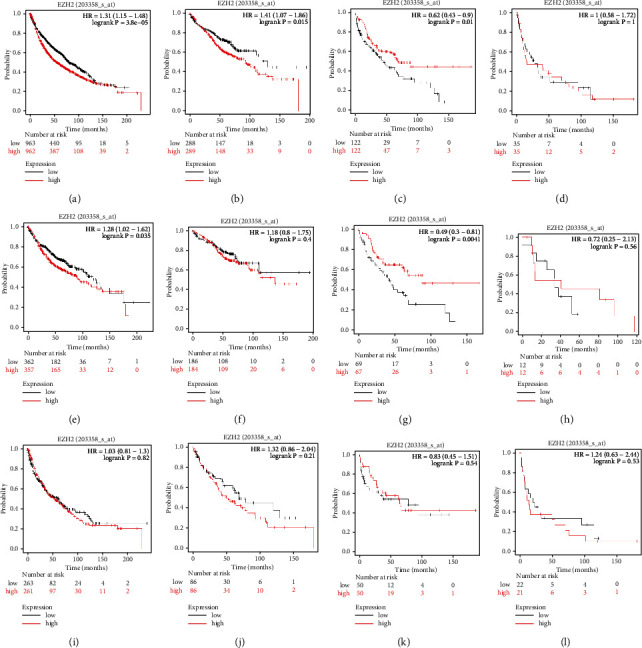
KM plotter validation of EZH2 expression in NSCLC patients. (a–c) Different OS in NSCLC and pTNM-stage I through II NSCLC with different EZH2 levels (all *P* < 0.05). (d) Different OS in pTNM-stage III NSCLC with different EZH2 levels (*P*=1). (e) Different OS in LUAD with different EZH2 levels (*P*=0.035). (f) Different OS in pTNM-stage I LUAD with different EZH2 levels (*P*=0.4). (g) Different OS in pTNM-stage II LUAD with different EZH2 levels (*P*=0.0041). (h) Different OS in pTNM-stage III LUAD with different EZH2 levels (*P*=0.56). (i–m) Different OS in all LUSC and pTNM-stage I through III LUSC with different EZH2 levels (all *P* > 0.05).

In order to testify the present findings, GEO, EGA, and TCGA data were used to verify the results found concerning RUNX3 and EZH2. As shown in Figures 7(a)–7(l), in the case of RUNX3, no statistical difference had been found in NSCLC with different pathologic stages or different histological types (all *P* > 0.05). However, in the case of EZH2 (Figures 8(a)–8(c)), higher expression was obviously correlated with worse outcome in NSCLC patients (*P* < 0.001), and the situation was the same in pathologic TNM I (*P*=0.015) and II (*P*=0.01) NSCLC patients, while no survival difference had been found in pTNM-III NSCLC patients (*P*=1, Figure 8(d)). In LUAD patients, however, higher EZH2 expression was correlated with worse outcome in all LUAD patients enrolled (*P*=0.035, Figure 8(e)) and in pTNM-II (*P*=0.0041, Figure 8(g)) while not in pTNM I or III patients (Figures 8(f)–8(h)). In LUSC patients, no correlation was determined (all *P* > 0.05, Figures 8(i)–8(m)).

**Figure 9:**
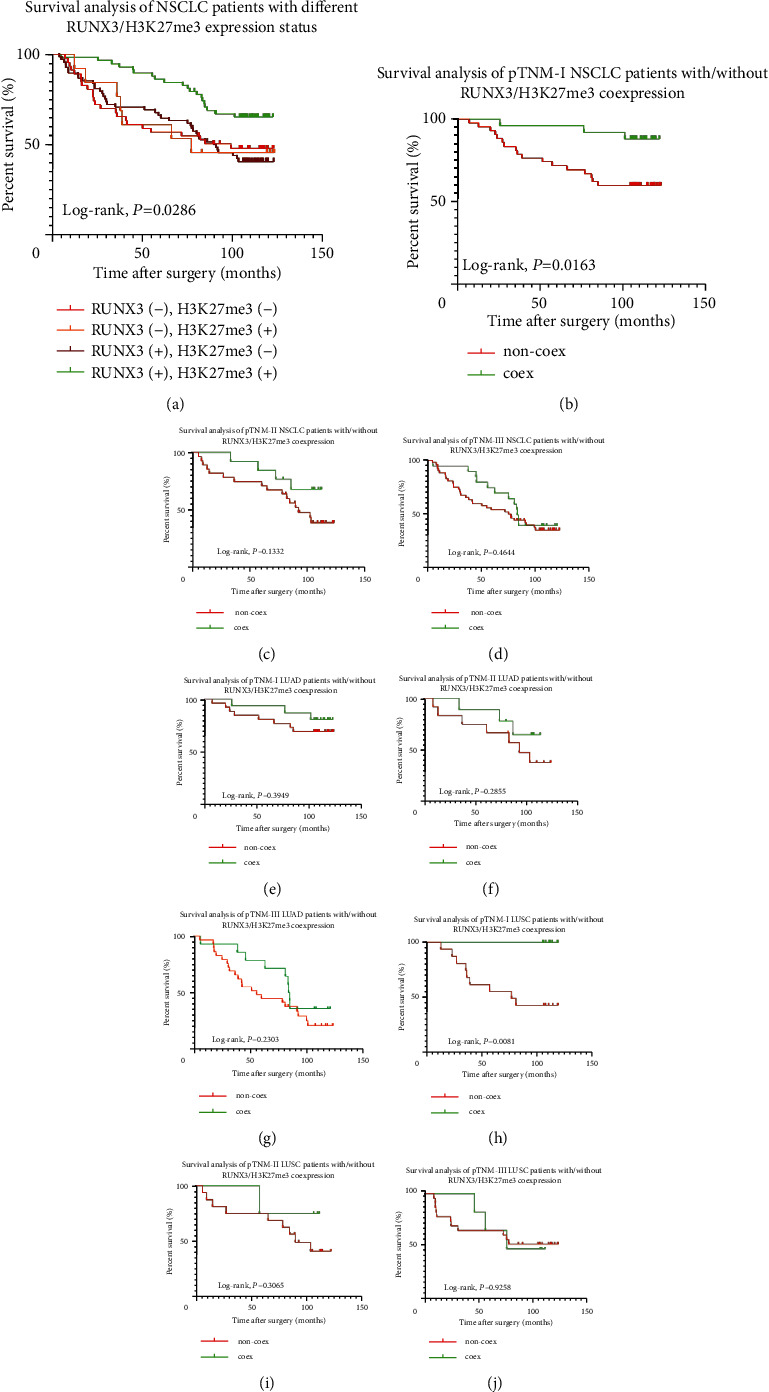
Survival analysis of the tested population. (a) Different OS in NSCLC patients with different RUNX3 and H3K27me3 status. (b) Different OS in pTNM-stage I NSCLC with different RUNX3/H3K27me3 co-expression status (*P*=0.0163). (c–d) Different OS in pTNM-stage II through III NSCLC with different RUNX3/H3K27me3 co-expression status (both *P* > 0.05). (e–g) Different OS in pTNM-stage I through III NSCLC with different RUNX3/H3K27me3 co-expression status (all *P* > 0.05). (h) Different OS in pTNM-stage I LUSC with different RUNX3/H3K27me3 co-expression status (*P*=0.0081). (i–j) Different OS in pTNM-stage II through III LUSC with different RUNX3/H3K27me3 co-expression status (both *P* > 0.05).

We then set out to analyze the survival curves in patients with concomitant expression of RUNX3/H3K27me3 and found out that, as shown in Figure 9(a), in the 4 subgroups based on different expression status of RUNX3/H3K27me3, NSCLC patients with RUNX3/H3K27me3 co-expression demonstrated the best OS in comparison to that of other subgroups, with either RUNX3 or H3K27me3 expression or neither. On this precondition, we further analyzed the outcome of NSCLC patients either with different pathologic staging or histology and found that patients with RUNX3/H3K27me3 co-expression exhibited better outcome and longer OS especially in the pTNM-I NSCLC (*P*=0.0163, *n* = 68) and pTNM-I LUSC (*P*=0.0081, *n* = 25) subgroups, while no survival difference had been demonstrated in other subgroups (all *P* > 0.05, Figures 9(b)–9(j)).

**Figure 10:**
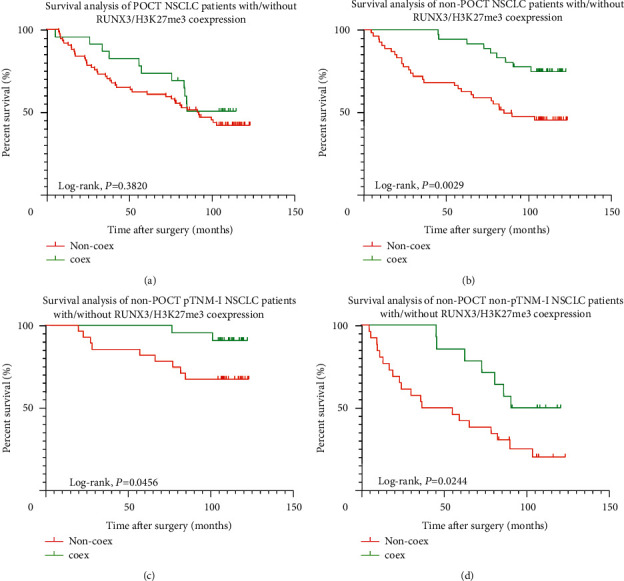
Survival analysis of population with POCT or non-POCT. (a) Different OS in POCT NSCLC patients with different RUNX3/H3K27me3 co-expression status (*P*=0.3820). (b) Different OS in non-POCT NSCLC patients with different RUNX3/H3K27me3 co-expression status (*P*=0.0029). (c) Different OS in non-POCT pTNM-stage I NSCLC patients with different RUNX3/H3K27me3 co-expression status (*P*=0.0456). (d) Different OS in non-POCT non-pTNM-stage I NSCLC patients with different RUNX3/H3K27me3 co-expression status (*P*=0.0244).

Adjuvant therapy, especially postoperative adjuvant chemotherapy (POCT), was often taken for patients with stage-II and above or in stage-I patients suffering postoperative relapse or metastasis. We wondered about the prediction efficacy that RUNX3/H3K27me3 co-expression might have on these populations and found that no survival difference had been determined in POCT patients (*P*=0.3820, Figure 10(a)) based on the different status of RUNX3/H3K27me3 co-expression, while in non-POCT patients (*P*=0.0029, Figure 10(b)), this difference was obvious, and non-POCT NSCLC patients with RUNX3/H3K27me3 co-expression demonstrated a better prognosis, and this situation was ever true in pTNM-I (*P*=0.0456, Figure 10(c)) and non-pTNM-I (*P*=0.0244, Figure 10(d)) patients.

### 3.5. Proliferative and Apoptotic Index

**Figure 11:**
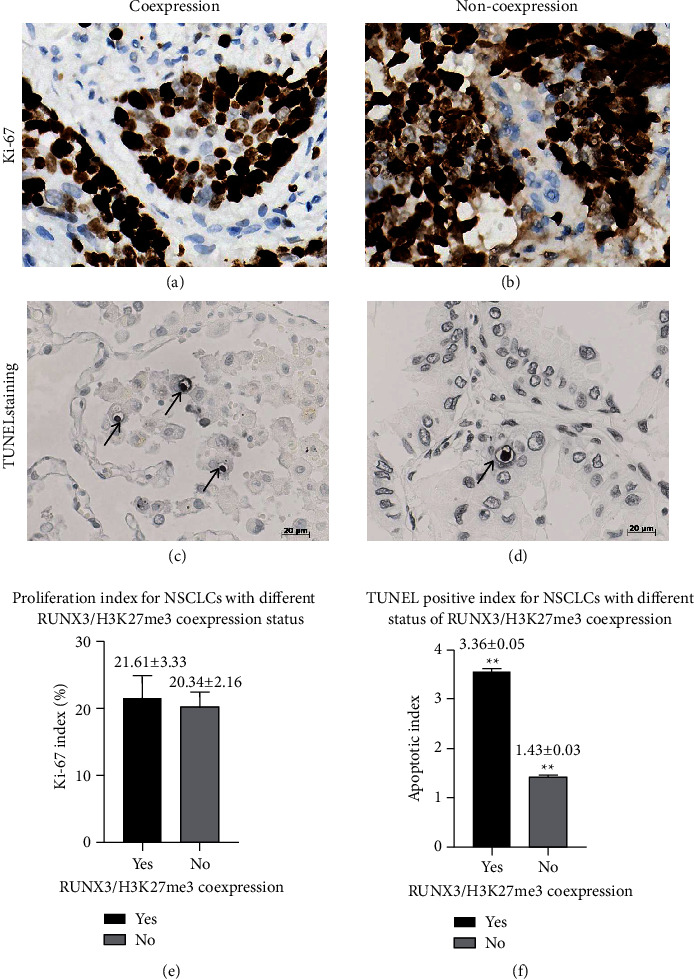
Cellular dynamic parameters between NSCLC patients with different RUNX3/H3K27me3 co-expression status. (a, b) Immunostaining for Ki-67 in NSCLC tissue with/without RUNX3/H3K27me3 co-expression. (c, d) TUNEL staining for apoptotic cells in NSCLC tissue with/without RUNX3/H3K27me3 co-expression, characterized with chromatin condensation and nuclear fragmentation, accompanied by pyknosis, retraction of pseudopodes, and formation of crescent caps of condensed chromatin at the nuclear periphery (arrows, 400× magnification, scale bar = 20 *μ*m). (e) Ki-67 index in NSCLC tissue with/without RUNX3/H3K27me3 co-expression (*P*=0.694). (f) Apoptotic index in NSCLC tissue with/without RUNX3/H3K27me3 co-expression (*P* < 0.001).

We then tried to further explore the underlying elements that might probably result in the different outcomes or OS in the facet of cellular proliferation and apoptosis from different co-expression status of RUNX3/H3K27me3 and found that no statistical difference had been demonstrated in the Ki-67 percentage between these two subgroups (Figures 11(a), 11(b), and 11(e), 21.61 ± 3.33 vs. 20.34 ± 2.16, *P*=0.694) while apoptotic index in the co-expression subgroup was statistically more than that in the non-co-expression subgroup (Figures 11(c), 11(d), and 11(f), 3.36 ± 0.05 vs. 1.43 ± 0.03, *P* < 0.001).

## 4. Discussion

Our previous findings [24–26] demonstrated the intimate connection between high H3K27me3 expression and low regional relapse, high RUNX3 level or nuclear localization of RUNX3, and low distant metastasis and hypothesized that the use of RUNX3/H3K27me3 co-expression would probably sensitize the prediction of both postoperative relapse and metastasis. In the present study, we investigated the prognostic value of RUNX3, H3K27me3, and EZH2 immunohistochemically in 188 surgically resected NSCLC patients, demonstrating that NSCLC patients with a cellular signature of simultaneous expressions RUNX3 and H3K27me3 would have better outcome and longer OS, irrespective of their histology and TNM staging. Further stratification analyses based on pathologic staging indicated that RUNX3/H3K27me3 co-expression might define some specific early-stage patient group (pTNM-I) so as to achieve good outcome and better survival after radical surgery and thereafter could be used as a good biomarker in the postoperative NSCLC patients. Surprisingly, in connection with postoperative regimens especially POCT, our findings still indicated that the prediction efficacy worked well especially in POCT-naive settings, irrespective of the histology or staging. In order to explain the phenomenon, we looked into the underlying mechanism resulting in the survival difference and we tried to compare the difference in cellular proliferation and apoptosis, finding out that co-expression subgroup demonstrates a higher apoptotic index in comparison to that of non-co-expression, while no difference had been determined in proliferation index. This could partially explain a better prognosis in the co-expression subgroup.

NSCLC patients with pTNM-I disease demonstrated a 5-year survival rate varying from 68% to 92% [8]. Early relapse and distant metastasis still remain a major concern in this patient population. Unlike locally advanced disease whose outcome might rely mainly on different comprehensive modalities, issues underlying early postoperative relapse/metastasis in early-stage patients, especially pTNM-I patients, might majorly contribute to the malignant behavior or genetic nature that tumor itself possesses. So, it is of vital importance to discriminate the “evil tumor” that would probably end up with worse outcome. Our present study concentrated on the basis to determine the factors affecting the outcome of surgically resected NSCLC patients. Consistent with our previous and others' findings, patients with good ECOG PS, no LN involvement, no mediastinal LN involvement, no metastasis at diagnosis, early disease (stage I/II), no pleural involvement, no lymphatic vessel involvement, no nerve involvement, R0 resection, postoperative radiotherapy, postoperative concurrent chemoradiotherapy, no CEA elevation at diagnosis, and no postoperative relapse or distant metastasis would have longer survival and better prognosis [30]. In addition, both Cox regression and Kaplan–Meier survival analysis with log-rank test confirmed that tumor with a signature of RUNX3/H3K27me3 co-expression exhibited a better prognosis in the survival analysis, especially in pathologic TNM-I population, which might help outline the high-risk population demanding close surveillance.

Postoperative therapy, either POCT or PORT, is often used in the locally advanced or postoperatively relapsed/metastasized settings. In our present study, about 52.1% (98/188) patients received POCT while around 19.7% (37/188) received PORT, and it is indicated by univariate analysis that POCT (*P*=0.089) did not help boost up OS in overall population while PORT (*P*=0.003) did. These findings were partially consistent with other reports [31–33]. Our further analysis on the effectiveness in the prediction of RUNX3/H3K27me3 co-expression among POCT or non-POCT population found that it worked only in non-POCT cohort, irrespective of the histological types or staging.

Putative tumor suppressor activity of RUNX3 has been presented extensively in many solid epithelial tumors, with loss of expression favoring tumorigenesis and/or prognosis [34]. However, this has been contradicted by other reports of RUNX3 behaving more like an oncogene, with overexpression leading to tumorigenesis [35]. Despite the controversy and inconsistent mechanistic evidence, the preponderance of evidence in the literature supports a role for RUNX3 in tumor biology and prognosis. In our previous study, it is indicated that loss of RUNX3 expression, irrespective of its localization, was an adverse factor related to the OS of NSCLC patients [26]. However, with the prolongation of follow-up time, no benefit had been demonstrated between patients subgroups with different level of RUNX3, as was also validated by TCGA dataset. In addition to its alteration in expression level, RUNX3 protein mislocalization from nucleus to cytoplasm was commonly reckoned as another risk factor leading to worse prognosis in cancer of stomach [36], colorectum [37], and breast [38], while not in lung cancer [26]. The mechanism underlying the translocation of RUNX3 protein in the abovementioned malignancies was thought to partially be correlated to factors like ATBF1 [39], src kinase [40–42], and TGF-*β* [43]. In the present study, however, we found that non-nuclear expression of RUNX3 was closely correlated to low level of EZH2 and H3K27me3 via immunohistochemistry, and enhanced RUNX3 expression was positively correlated with high level of EZH2 and H3K27me3, as was also confirmed via fluorescent double immunostaining and western blot. It was reported by Fujii et al. [9] that EZH2 downregulated RUNX3 by increasing H3K27me3 in gastric, breast, prostate, colon, and pancreatic cancer cell lines. However, Rehman et al. [44] recently reported a significant positive correlation between RUNX3 and EZH2 in Indian patients with esophageal cancer, which was similar to our finding in NSCLC. This paradox results concerning RUNX3 might inevitably point to the debate that whether RUNX3 functions as a tumor suppressor gene or oncogene or even both were depending on specific tumor context. Recent exploration indicated that whether RUNX3 played a role of TSG or oncogene was dependent on the status of p53 [45]. That is to say, in the state of DNA damage or oncogenic stress, RUNX3 would positively regulate p53 and was in turn suppressed by it, and p53 would prevent tumorigenesis by decreasing the activity of some crucial oncogenes and thus retard the onset of malignancy. Upon inactivation of p53, dysregulated RUNX3 started to upregulate MYC aberrantly. Thus, p53 status was considered as a contextual determinant for whether RUNX3 behaved as a tumor suppressor or oncogene. Our further study in future might take these issues into account and try to figure out the mechanism underlying the balance of between the roles that RUNX3 might play as an oncogene or TSG.

Some limitation in this study should be mentioned. Firstly, the sample size used in our study is relatively small, and a cohort with a larger sample size to test the authenticity would be necessary. Secondly, this is a retrospective study and only overall survival rather than progression-free survival was taken into account, so the results achieved in the present study would need a validation by a prospective study with both PFS and OS in the future. Thirdly, intense study focusing on the interaction and mechanism of RUNX3/H3K27me3 co-expression should be further undermined.

## 5. Conclusion

RUNX3/H3K27me3 co-expression defined some specific pTNM-I NSCLC population with higher proportion of apoptotic index and thus better prognosis and longer OS and could probably be used as a biomarker in the prediction of postoperative relapse and metastasis.

## Figures and Tables

**Table 1:** List of antibodies used in immunohistochemistry.

Antibodies	Manufacturer	Catalogue no.	Clonality/clone no.	Working dilution
Mouse anti-human RUNX3	Abcam, Cambridge, UK	Ab135248	Monoclonal/2B3	1 : 500
Rabbit anti-human H3K27me3	Cell Signaling Technology, MA, USA	#9733	Polyclonal/C36B11	1 : 200
Rabbit anti-human EZH2	Cell Signaling Technology, MA, USA	#5246	Monoclonal/30-9	1 : 400
Rabbit anti-human Ki-67	Roche Applied Science (Penzberg, Germany)	—	Monoclonal/30-9	Instant use
HRP-conjugated goat anti-rabbit/mouse IgG	Millipore Co, CA, USA	—	—	1 : 200
Normal goat IgG	Sigma Chemical Co, MO, USA	—	—	1 : 20
Alexa 488 anti-mouse/Alexa546 anti-rabbit	Invitrogen Co, CA, USA	—	—	1 : 500
FITC-labeled goat anti-biotin	Vector Laboratories, CA, USA	—	—	1 : 100
Rhodamine-labeled sheep anti-digoxigenin	Roche Diagnostics, Mannheim, Germany	—	—	1 : 100

**Table 2:** Clinicopathologic parameters of patients.

Parameters	Number of cases (%)	
	LUAD	LUSC
Median age (y.o.)	57.5	59.0
Median follow-up (mon)	79.5	81.0
Age (y.o.)		
≤58	64 (56.6)	39 (52.0)
>58	49 (43.4)	36 (48.0)
Gender		
Male	63 (55.8)	65 (86.7)
Female	50 (44.2)	10 (13.3)
Histology	113 (60.1)	75 (39.9)
Differentiation		
Well	NA	4 (5.3)
Moderately	NA	57 (76.0)
Poorly	NA	14 (18.7)
Predominant growth pattern		
Lepidic	10 (8.8)	NA
Acinar	75 (66.4)	NA
Papillary	10 (8.8)	NA
Micropapillary	1 (1.0)	NA
Solid	17 (15.0)	NA
BMI (kg/m^2^)		
≤20.3	17 (9.0)	13 (6.9)
>20.3	96 (51.1)	62 (33.0)
ECOG PS		
≤1	86 (76.1)	59 (78.7)
>1	27 (23.9)	16 (21.3)
Smoker		
Yes	56 (49.6)	60 (80.0)
No	57 (50.4)	15 (20.0)
sCEA (ng/ml)		
<4.7	58 (51.3)	56 (74.7)
≥4.7	55 (48.7)	19 (25.3)
PI		
Yes	92 (81.4)	44 (58.7)
No	21 (18.6)	31 (41.3)
VI		
Yes	13 (11.5)	4 (5.3)
No	100 (88.5)	71 (94.7)
LVI		
Yes	54 (47.8)	31 (41.3)
No	59 (52.2)	44 (58.7)
NI		
Yes	45 (39.8)	24 (32.0)
No	68 (60.2)	51 (68.0)
RUNX3 localization		
Negative	35 (31.0)	20 (26.7)
Nuclear	3 (2.7)	7 (8.9)
Cytoplasm	38 (33.6)	14 (18.7)
Whole-cell	37 (32.7)	34 (45.3)
pTNM staging		
I	43 (38.1)	25 (33.3)
II	21 (18.6)	20 (26.7)
III	43 (38.1)	30 (40.0)
IV	6 (5.2)	0 (0)
T-staging		
1a	1 (0.9)	1 (1.2)
1b	7 (6.2)	6 (8.0)
1c	13 (11.5)	5 (6.7)
2a	58 (51.3)	24 (32.0)
2b	15 (13.3)	8 (10.7)
3	12 (10.6)	14 (18.7)
4	7 (6.2)	17 (22.7)
N-staging		
0	54 (47.8)	43 (57.3)
1	17 (15.0)	15 (20.0)
2	31 (27.4)	15 (20.0)
3	11 (9.8)	2 (2.7)
M-staging		
0	107 (94.7)	75 (100)
1a	3 (2.7)	0 (0)
1b	2 (1.8)	0 (0)
1c	1 (0.8)	0 (0)
Surgery		
Sublobar resection	5 (4.4)	0
Lobectomy	92 (81.4)	52 (69.3)
Combined lobectomies	12 (10.6)	12 (16.0)
Pneumonectomy	4 (3.6)	11 (14.7)
VATS		
Yes	52 (46.0)	13 (17.3)
No	61 (54.0)	62 (82.7)
PORT		
Yes	23 (20.4)	14 (18.7)
No	90 (79.6)	61 (81.3)
POCT		
Yes	57 (50.4)	41 (54.7)
No	56 (49.6)	34 (45.3)
POCRT		
Yes	19 (16.8)	13 (17.3)
No	94 (83.2)	62 (82.7)
Relapse		
Yes	27 (23.9)	20 (26.7)
No	86 (76.1)	55 (73.3)
Metastasis		
Yes	43 (38.1)	24 (32.0)
No	70 (61.9)	51 (68.0)

**Table 3:** Associations of RUNX3, H3K27me3, and EZH2 expression with clinicopathologic parameters in NSCLC patients.

Parameters	RUNX3				H3K27me3				EZH2			
	All	*H*	*L*	*P*, *P* value	All	*H*	*L*	*P*, *P* value	All	*H*	*L*	*P* value
Age^a^ (y.o.)				0.025				0.640				0.638
≤58	103	63	40		103	41	62		103	33	70	
>58	85	65	20		85	31	54		85	30	55	
Gender				0.196				0.010				0.714
Female	60	37	23		60	31	29		60	19	41	
Male	128	91	37		128	41	87		128	44	84	
Histology				0.536				0.148				0.005
LUAD	113	75	38		113	48	65		113	29	84	
LUSC	75	53	22		75	24	51		75	34	41	
ECOG PS				0.019				<0.001				0.341
≤1	145	105	40		145	66	79		145	46	99	
>1	43	23	20		43	6	37		43	17	26	
BMI^b^ (kg/m^2^)				0.059				0.841				0.386
≤20.3	30	16	14		30	11	19		30	8	22	
>20.3	158	112	46		158	61	97		158	55	103	
Smoker				0.995				<0.001				0.720
Yes	116	79	37		116	40	84		116	40	76	
No	72	49	23		72	32	32		72	23	49	
LVI				0.223				0.094				0.881
Yes	85	54	31		85	27	58		85	28	57	
No	103	74	29		103	45	58		103	35	68	
NI				0.197				0.168				0.719
Yes	69	43	26		69	22	47		69	22	47	
No	119	85	34		119	50	69		119	41	78	
PI				0.364				0.521				0.843
Yes	136	90	46		136	54	82		136	45	91	
No	52	38	14		52	18	34		52	18	34	
VI				0.588^c^				0.068				0.483
Yes	17	13	4		17	10	7		17	7	10	
No	171	115	56		171	62	109		171	56	115	
T-staging				0.488				0.159				0.663
T1-2	138	92	46		138	57	81		138	45	93	
T3-4	50	36	14		50	15	35		50	18	32	
N-staging-1				0.062				0.079				0.642
N0	97	72	25		97	43	54		97	31	66	
N1–3	91	56	35		91	29	62		91	32	59	
N-staging-2				0.464				0.401				0.939
N0-1	129	90	39		129	52	77		129	43	86	
N2-3	59	38	21		59	20	39		59	20	39	
M-staging				0.083^c^				0.409^c^				0.665^c^
M0	182	126	56		182	71	111		182	62	120	
M1	6	2	4		6	1	5		6	1	5	
TNM staging-1				0.230				0.110				0.882
I-II	109	78	31		109	47	62		109	37	72	
III-IV	79	50	29		79	25	54		79	26	53	
TNM staging-2				0.579				0.030				0.800
I	68	48	20		68	33	35		68	22	46	
II–IV	120	80	40		120	39	81		120	41	79	
Resectibility				0.788				0.584				0.341
R0	145	98	47		145	54	91		145	46	99	
R1-2	43	30	13		43	18	25		43	17	26	
sCEA (*μ*g/ml)				0.160				0.305				0.376
≤4.7	114	82	32		114	47	67		114	41	73	
>4.7	74	46	28		74	25	49		74	22	52	
Relapse				0.148				0.006				0.129
Yes	47	28	19		47	10	37		47	20	27	
No	141	100	41		141	62	79		141	43	98	
Metastasis				0.002				0.252				0.639
Yes	67	36	31		67	22	45		67	21	46	
No	121	92	29		121	50	71		121	42	79	
RUNX3^b^				—				0.001				0.018
≤3	—	—	—		60	13	47		56	12	44	
>3	—	—	—		128	59	69		132	51	81	
RUNX3 location				—				0.003				0.001
Nuclear	—	—	—		81	41	40		81	38	43	
Non-nuclear	—	—	—		107	31	76		107	25	82	
H3K27me3^b^				0.001				—				0.002
≤4	116	69	47		—	—	—		100	29	87	
>4	72	59	13		—	—	—		88	34	38	
EZH2^a^				0.018				0.002				—
≤1.9	125	78	47		125	38	87		—	—	—	
>1.9	63	50	13		63	34	29		—	—	—	
KI-67 (%)				0.701				0.404				<0.001
≤10	109	73	36		109	39	70		109	13	96	
>10	79	55	24		79	33	46		79	50	29	

*Note*. All, all cases; *H*, high expression; *L*, low expression; *p*^a^average value; ^b^cutoff point determined by X-tile software; ^c^Fisher's exact test (two-sided); *χ*^2^ test for all other analyses.

**Table 4:** Associations of RUNX3/H3K27me3 co-expression status with clinicopathologic parameters in NSCLC patients.

Parameters	All	Yes	No	*χ* ^2^	*P* value
Age^a^ (y.o.)				0.010	0.918
≤58	103	32	71		
>58	85	27	58		
Gender				4.328	0.037
Female	60	25	35		
Male	128	34	94		
Histology				2.121	0.145
LUAD	113	40	73		
LUSC	75	19	56		
ECOG PS				NA	<0.001^c^
≤1	145	56	89		
>1	43	3	40		
BMI^b^ (kg/m^2^)				1.074	0.300
≤20.3	30	7	23		
>20.3	158	52	106		
Smoker				11.315	0.001
Yes	116	26	90		
No	72	33	39		
LVI				1.347	0.246
Yes	85	23	62		
No	103	36	67		
NI				0.749	0.387
Yes	69	19	50		
No	119	40	79		
PI				0.057	0.811
Yes	136	42	94		
No	52	17	35		
VI				2.133	0.144
Yes	17	8	9		
No	171	51	120		
T-staging				1.724	0.189
T1-2	138	47	91		
T3-4	50	12	38		
N-staging-1				2.055	0.152
N0	97	35	62		
N1–3	91	24	67		
N-staging-2				0.264	0.608
N0-1	129	42	87		
N2-3	59	17	42		
M-staging				NA	0.667^c^
M0		58	124		
M1		1	5		
TNM staging-1				1.458	0.227
I-II	109	38	71		
III-IV	79	21	58		
TNM staging-2				1.433	0.231
I	68	25	43		
II–IV	120	34	86		
Resectibility				0.036	0.850
R0	145	45	100		
R1-2	43	14	29		
sCEA (*μ*g/ml)				1.846	0.174
≤4.7	114	40	74		
>4.7	74	19	55		
Relapse				12.523	<0.001
Yes	47	5	42		
No	141	54	87		
Metastasis				3.911	0.048
Yes	67	15	52		
No	121	44	77		
RUNX3				—	—
≤3	—	—	—		
>3	—	—	—		
RUNX3 localization				24.450	<0.001
Nuclear	81	41	40		
Non-nuclear	107	18	89		
EZH2^a^				5.793	0.016
≤1.9	125	32	93		
>1.9	63	27	36		
Ki-67				0.004	0.947
≤10%	109	34	75		
>10%	79	25	54		

*Note*. All, all cases; Yes, co-expression; No, non-co-expression; *p*^a^average value; ^b^cutoff point determined by X-tile software; ^c^Fisher's exact test (two-sided); *χ*^2^ test for all other analyses (two-sided).

**Table 5:** Univariate analysis of factors related to overall survival.

Parameters	HR (95% CI)	*P* value
Age (≤58 y.o. vs. >58 y.o.)	0.972 (0.643–1.467)	0.891
Gender (male vs. female)	0.771 (0.492–1.210)	0.258
Smoker (yes vs. no)	0.802 (0.523–1.230)	0.312
ECOG PS (≤1 vs. >1)	0.016 (0.008–0.033)	<0.001
BMI-2 (≤20.3 vs. >20.3)	1.415 (0.824–2.429)	0.208
Histology (LUSC vs. LUAD)	0.847 (0.552–1.298)	0.445
T-staging (T1-2 vs. T3-4)	0.741 (0.474–1.156)	0.187
N-staging-1 (N0 vs. N1–3)	0.468 (0.307–0.716)	<0.001
N-staging-2 (N0-1 vs. N2-3)	0.412 (0.272–0.625)	<0.001
M-staging (M0 vs. M1)	0.164 (0.071–0.382)	<0.001
TNM-1 (I-II vs. III-IV)	0.431 (0.284–0.653)	<0.001
TNM-2 (I vs. II–IV)	0.389 (0.236–0.639)	<0.001
PI (yes vs. no)	0.508 (0.300–0.862)	0.012
LVI (yes vs. no)	0.527 (0.347–0.799)	0.003
VI (yes vs. no)	0.720 (0.373–1.392)	0.329
NI (yes vs. no)	0.471 (0.312–0.712)	<0.001
Resectibility (R0 vs. R1-2)	0.404 (0.261–0.625)	<0.001
PORT (yes vs. no)	0.496 (0.314–0.782)	0.003
POCT (yes vs. no)	0.696 (0.459–1.056)	0.089
POCRT (yes vs. no)	0.506 (0.313–0.819)	0.006
sCEA (≤4.7 vs. >4.7)	0.496 (0.328–0.749)	0.001
Relapse (yes vs. no)	0.024 (0.012–0.045)	<0.001
Metastasis (yes vs. no)	0.222 (0.144–0.341)	<0.001
RUNX3 (high vs. low)	1.301 (0.843–2.008)	0.235
Nuclear RUNX3 (yes vs. no)	1.185 (0.780–1.800)	0.428
H3K27me3 (high vs. low)	1.815 (1.157–2.847)	0.009
EZH2 (high vs. low)	0.958 (0.619–1.484)	0.849
RUNX3/H3K27me3 co-expression (no vs. yes)	2.099 (1.277–3.450)	0.003

**Table 6:** Multivariate analyses of factors related to overall survival.

Parameters	HR (95% CI)	*P* value
M-staging	0.599 (0.381–0.943)	0.027
TNM staging (I vs. II–IV)	0.758 (0.584–0.982)	0.036
Relapse (yes vs. no)	0.322 (0.208–0.499)	<0.001
ECOG PS (≤1 vs. >1)	0.347 (0.227–0.531)	<0.001
Metastasis (yes vs. no)	0.762 (0.585–0.992)	0.044

## Data Availability

The dataset of the public database can be found from the following websites: GEPIA (cancer-pku.cn); Kaplan-Meier plotter [Lung] (kmplot.com).

## Abbreviations

NSCLC: Non-small-cell lung cancer
RUNX3: Runt-related transcription factor 3
H3K27me3: Trimethylated histone H3 at lysine 27
EZH2: Enhancer of zeste homolog 2
TMA: Tissue microarray
TUNEL: TdT mediated dUTP-biotin nick end labeling
LUSC: Lung squamous-cell carcinoma
LUAD: Lung adenocarcinoma
GEPIA: Gene expression profiling interactive analysis
ECOG PS: Eastern cooperative oncology group performance score
NA: Not applicable
HR: Hazard ratio
CI: Confidence interval
BMI: Body mass index
LVI: Lymphatic vessel invasion
NI: Nerve invasion
PI: Pleural invasion
VI: Vascular invasion
sCEA: Serum carcinoembryonic antigen
VATS: Video-assisted thoracic surgery
POCT: Postoperative chemotherapy
PORT: Postoperative radiotherapy
POCRT: Postoperative chemoradiotherapy.
